# Unparalleled details of soft tissues in a Cretaceous ant

**DOI:** 10.1186/s12862-022-02099-2

**Published:** 2022-12-16

**Authors:** Yuhui Zhuang, Wenjing Xu, Guojie Zhang, Huijuan Mai, Xiaoqin Li, Hong He, Hao Ran, Yu Liu

**Affiliations:** 1 Yunnan Key Laboratory for Palaeobiology, Institute of Palaeontology, Yunnan University, South Waihuan Road, Chenggong District, Kunming, 650500 China; 2 MEC International Joint Laboratory for Palaeobiology and Palaeoenvironment, Yunnan University, Kunming, 650500 China; 3 Key Laboratory of National Forestry and Grassland Administration On Management of Forest Bio-Disaster, College of Forestry, Northwest A&F University, Yangling, 712100 Shaanxi China; 4 Evolutionary & Organismal Biology Research Center, Zhejiang University School of Medicine, Hangzhou, 310058 China; 5 State Key Laboratory of Genetic Resources and Evolution, Kunming Institute of Zoology, Chinese Academy of Sciences, Kunming, 650223 China; 6 Villum Center for Biodiversity Genomics, Section for Ecology and Evolution, Department of Biology, University of Copenhagen, Copenhagen, Denmark; 7 Key Laboratory of Ecology of Rare and Endangered Species and Environmental Protection, Ministry of Education, Guilin, 541004 China; 8 Biological Education and Research Laboratory, Mancheng High School of Hebei Province, Baoding, 072150 China

**Keywords:** Amber, Cretaceous, Fossil, Micro-CT, Soft-tissue preservation, *Zigrasimecia*

## Abstract

For social insects such as ants, the internal organs are likely important in understanding their eusocial behavior and evolution. Such organs, however, are rarely preserved on fossils. In each of the few cases reporting exceptionally fossilized soft tissues in arthropods, the nervous, muscular and cardiovascular systems have been described individually, but never in combination. Here, we report a female specimen (gyne) of the extinct ant group—^†^*Zigrasimecia*—included in a Cretaceous amber piece from Kachin, Myanmar, with an almost complete system formed by various internal organs. These include the brain, the main exocrine system, part of the digestive tract, and several muscle clusters. This research expands our knowledge of internal anatomy in stem group ants. As the gyne bears a morphologically unique labrum, our specimen’s internal and external features support the notion that the early ant may have special ecological habits during the Cretaceous period.

**Supplementary Information:**

The online version contains supplementary material available at 10.1186/s12862-022-02099-2.

## Background

With a species number of more than 14,000, ants represent one of the dominant groups of eusocial insects and survive in almost all terrestrial ecosystems of this planet [1, 2]. The evolution of ants and the origin of their eusocial behavior are intriguing not only for entomologists but also the general public. To address these topics, biologists have carried out morphological [1], ethological [1, 2], and molecular [3–6] studies on living ants. Phylogenetic analyses based on large-scale molecular data sets have suggested that ants evolved from wasp-like ancestors at least 115–135 million years ago [4–6]. Ants have been considered as the first group of the ground-dwelling predatory eusocial insects [7]. The sociality of ants is generally thought to be empowered by a complex nervous system [8, 9] and chemical pheromones [7, 10, 11]. On the one hand, the social behaviour of ants has been reported from Cretaceous amber pieces of Kachin [12]. On the other hand, neural and other soft tissues that enable ants’ sociality remains poorly known from the fossils. The few examples include a gland reported by Brady and colleagues based on their microscopic observations [3], and the nerve and muscle tissues discovered by Boudinot and his colleagues in a Cretaceous amber piece [13, 14].

In general, internal organs are rarely preserved on fossils due to decay and compaction processes during fossilization [15]. Compared to other types of fossil preservation, ambers provide a better chance for soft tissues to be preserved and observed [16]. Examples of soft tissue preservation in Kachin ambers include muscles of a wasp [17] and a beetle [18], giant sperms of an ostracod [19], and the digestive duct of a shrimp [20]. An extreme case is the full-body 3D reconstruction including the brain, muscles, and sperm pump of the extinct group—†Mengea (stem group of Strepsiptera)—from a Baltic amber [21].

Here, we report our findings in a female specimen (gyne) of the extinct ant group—^†^*Zigrasimecia*—in a Cretaceous amber piece from Kachin, with exceptionally preserved and almost complete internal organs such as the brain, the main exocrine system, the digestive tract, and several muscle clusters. ^†^*Zigrasimecia* belongs to the subfamily ^†^Zigrasimeciinae which was considered as one of the oldest ant subfamilies [22–27]. Our findings, therefore, not only expand the knowledge of fossil ants to their internal anatomy but also shed new lights on understanding the early evolution of ants and their early sociality.

## Materials and methods

### Studied material

**Fig. 1:**
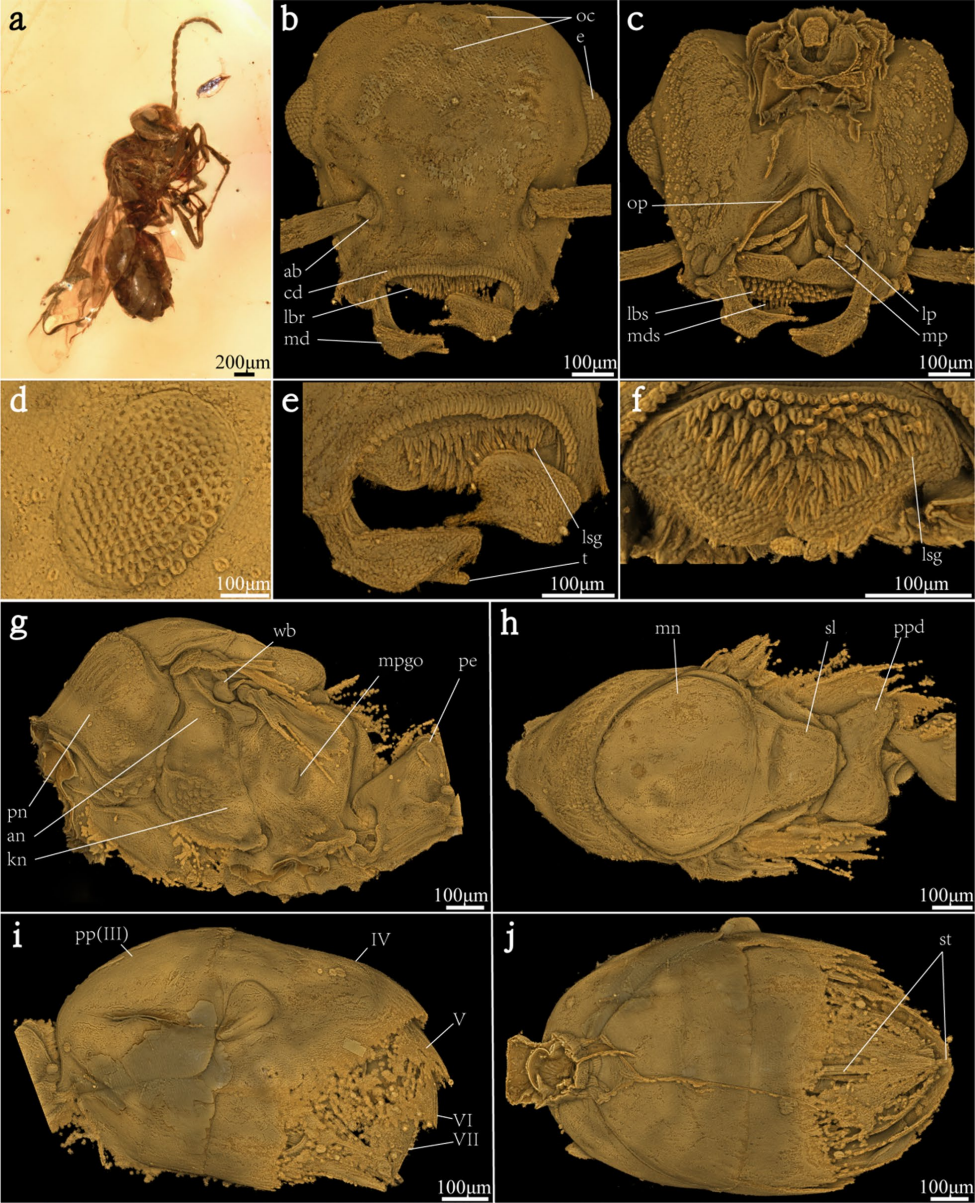
Specimen (YKLP-AMB-002) and 3D reconstructions of †*Zigrasimecia* sp. **a** Specimen in amber. **b** Head in anterior view. **c** Head in posterior view. **d** Left compound eye. **e** Close-up of mouth part. **f** Close-up of labrum with labial setae each bearing a groove. **g**, **h** Mesosoma in lateral and dorsal views. **i**, **j** Gaster in lateral and bottom views. *ab* antennal base; *an* anepisternum; *cd* clypeal denticles; *kn* katepisternum; *lbr* labrum; *lbs* labial setae; *lp* labial palp; *lsg* labial setae groove; *md* mandible; *mds* mandibular setae; *mn* mesonotum; *mp* maxillary palp; *mpgo* metapleural gland opening; *op* oral opening; *pe* petiole; *pn* pronotum; *pp* postpetiole; *ppd* propodeum; *sl* scutellum; *st* sting; *t* tooth; *wb* wing base (III–VII, third through seventh abdominal segments)

Amber samples. Specimen No. YKLP-AMB-002. A gyne trapped in a piece of nearly transparent and yellow amber (Fig. 1a), which was collected by Wanglin Zheng in Noije Bum Village, Danai Town, Kachin Province, Myanmar. The dimensions of the piece are 24.7 mm (length), 18.8 mm (width) and 4.3 mm (height). The weight of the piece is 1.75 g. The U–Pb isotope dating age of this Kachin amber is 98.79 ± 0.62 Ma [28]. Yu and Wang have reaffirmed this dating result by trapped ammonite and ostracods in the amber [19, 29]. The amber (YKLP-AMB-002) is deposited at the Yunnan Key Laboratory for Palaeobiology (YKLP), Institute of Palaeontology, Yunnan University, Kunming, China. Structure termilogy refer from Bolton, 1994 [30].

### Micro-CT scanning

The specimen was scanned at the micro-CT laboratory of YKLP with an X-ray microscope (3D-XRM), Zeiss Xradia 520 versa, and divided into three parts. Scanning parameters are as the following: YKLP-AMB-002 (head): Beam strength: 60 kV/5w, Filter: no, Resolution: 0.97 µm, Exposure time: 5 s, Number of TIFF images: 986; YKLP-AMB-002 (mesosoma): Scanning parameters are as the following: Beam strength: 60 kV/5w, Filter: no, Resolution: 0.94 µm, Exposure time: 5.5 s Number of TIFF images: 1617; YKLP-AMB-002 (gaster): Scanning parameters are as the following: Beam strength: 60 kV/5w, Filter: no, Resolution: 1.15 µm, Exposure time: 4.5 s, Number of TIFF images: 992. Volume rendering and 3D reconstruction were performed using the open-source software Drishti 2.4 [31]. For shooting and super depth of field synthesis of the surface characteristics of the whole amber, a Keyence VHX-6000 3D microscope was used. Figures were processed in Photoshop 2019 and Illustrator 2020 (Adobe Systems Incorporated, San José, USA).

## Results

### Systematic palaeontology

Subfamily ^†^Zigrasimeciinae Borysenko et al*.*, 2017.

Genus ^†^*Zigrasimecia* Barden et al*.*, 2013.

**Species** ^†^*Zigrasimecia* sp.

**Material** YKLP-AMB-002, gyne (Fig. 1a).

**Type locality and stratigraphy** Hukawng Valley, Kachin Province, northern Myanmar. Upper Albian-lower Cenomanian (ca. 98.79 ± 0.62 Ma).

### External morphology

Head (Fig. 1b–f): wide and flattened in face view (Fig. 1b), oval-shape in lateral view. Oval-shaped small compound eyes (Fig. 1b, d). Three ocelli small (Fig. 1b). Antenna 12 flagellomeres (Fig. 1a). Lateromedially broad clypeus with concave anterior margin, lateral edges curved downward and forming convex lobes covering the mandibular bases; anterior clypeal margin with 36 clypeal denticles, which closely arranged, shortening from middle to sides. Labrum large, with 98 labral setae (Fig. 1e). Upper and middle labral setae are longer than lateral and lower setae, each with distinct groove above. The deepest depth of labral setae’s groove about one-third of setae's diameter (Fig. 1f). The lowermost part of labrum densely covered with short, soft setae. Hypostomal carina deeply V-shaped in ventral view, hypostomal cavity thus rather open, giving space for the labiomaxillary complex to move. Mandibles barely overlap each other, with two teeth on chewing side, apex of subapical tooth aligning with apical tooth; inner edge covered with hard and sharp needle-like setae, each mandibular seta can insert into a root of labral setae groove. Maxillary palp 3-merous. Labial palp 4-merous.

Mesosoma (Fig. 1g, h): Developed. Neck short, almost completely covered by pronotum. In lateral and dorsal views, junctions between each notum well developed. Pronotum well developed and wider than mesonotum. Anepisternum and katepisternum well differentiated from each other by grooves. Scutellum thick in dorsal view. Propodeum concaved in the middle and rear side wider than front side, developing posteriorly towards the body. Petiole one segment. Dorsal surface of the petiole is slightly concave.

Gaster (Fig. 1i, j): with five segments and distinctly broader than head and mesosoma, about one-half of body length. The first gastral segment large in lateral view, measuring about one-third of gastral length. The second gastral segment distinctly longer than other segments in dorsal view, about one-half of gastral length. Third gastral segment longer than the fourth and comparable in length to the fifth. Exposed part of sting short in length, but the sting inner part about two thirds of gaster length.

Measurements (in μm): head (including eyes) length 492.6, width 765.2; ocelli length 33.3, width 22.1; compound eyes length 212.8, width 169.7; clypeal denticles length 12.0–26.0; labrum length 135.9, width 317.9; labral setae longest length 30.3, shortest length 15.7; mandible length 309.2; oral opening length 199.8, width 285.7. Mesosoma length 829.1, width 432.2 and height 640.8; petiole length 292.0, height 368.7. Gaster length 1084.2, width 621.6, height 640.5.

### Nervous system

**Fig. 2:**
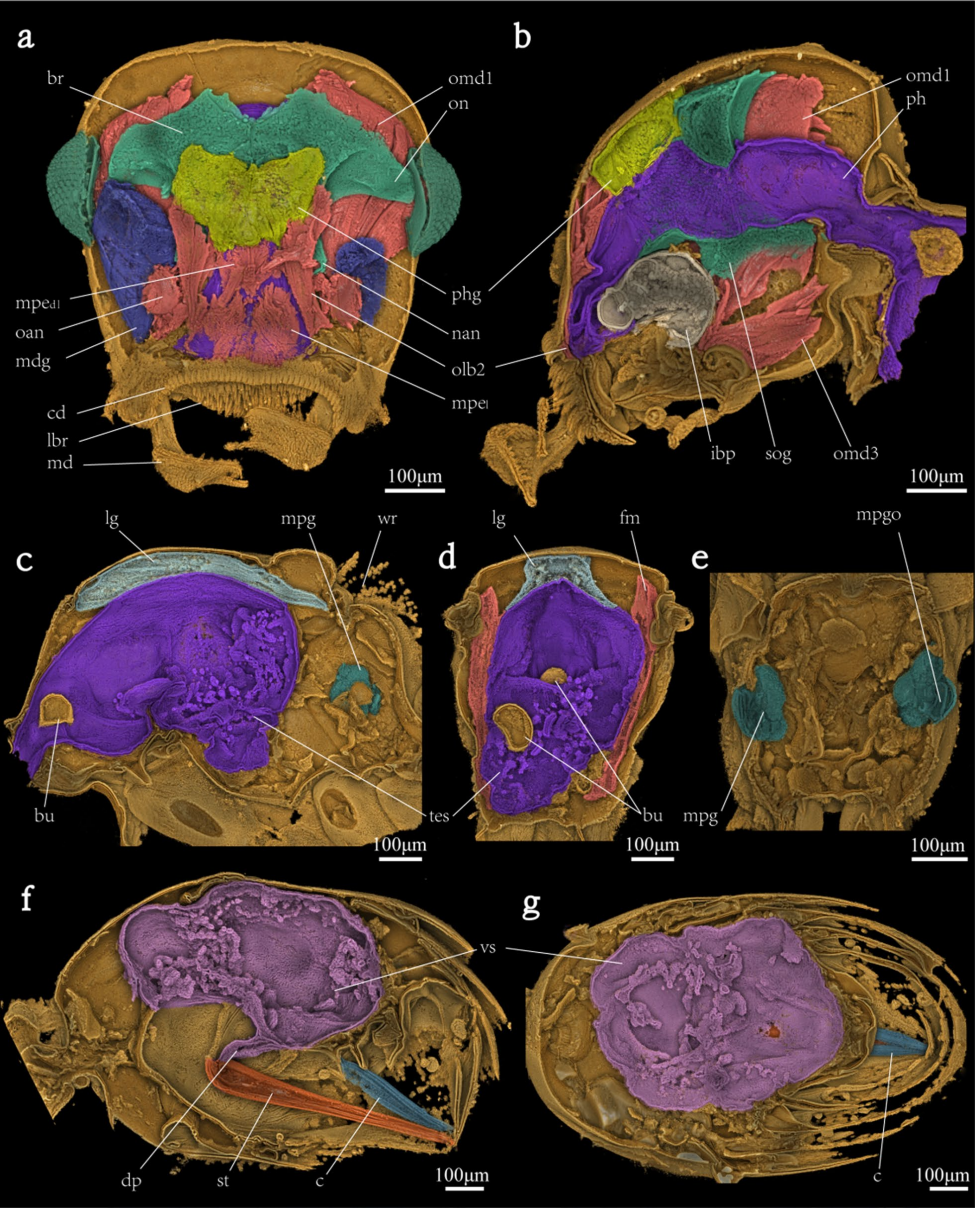
Anatomy of †*Zigrasimecia* sp. (YKLP-AMB-002). **a**, **b** Head anatomy in anterior and lateral views. **c** Longitudinal section showing mesosoma anatomy in lateral view. **d** Cross section of mesosoma showing mesosoma anatomy in dorsal view. **e** Cross section of mesosoma showing metapleural gland and its opening. **f** Longitudinal section showing gaster anatomy in lateral view. **g** Horizontal section showing gaster anatomy in top view. *br* brain; *bu* bubble; *c* cloacal chamber; *cd* clypeal denticles; *dp* main duct of poison sac; *fm* flying muscle; *ibp* infrabuccal pocket; *lbr* labrum; *lg* labial gland; *md* mandible; *mdg* mandibular gland; *mpe*_*dl*_ M. pharyngoepipharyngalis lateral dorsal portion; *mpe*_*l*_ M. pharyngoepipharyngalis lateral portion; *mpg* metapleural gland; *mpgo* metapleural gland opening; *nan* antennal nerve; *omd1* M. craniomandibularis internus; *omd3* M. craniomandibularis externus; *oan* M. tentorioscapalis; *olb2* M. frontoepipharyngalis; *on* optical nerve; *ph* pharynx; *phg* pharyngeal gland; *st* sting; *sog* suboesophageal ganglion; *tes* thoracic esophagus; *vs* venom gland sac; *wr* wing remain

In the head of YKLP-AMB-002, a sub-reniform-shaped organ is preserved. It is located at the center of the head and bears connecting structures to the compound eyes and antenna (Fig. 2a, b). Based on its shape and position, it can be determined as the nerve center, or brain, of YKLP-AMB-002 [32–34]. Although the preservation of the fossil may be subjected to deformation and shrinkage, the brain is well developed (width: 557 μm, height: 341 μm, maximum thickness: 241 μm, i.e., 49% of the head length). The brain is connected to the subesophageal complex to form a compact unit, connected by neurons on both sides of the oesophagus. The optic nerve is thick in frontal view, connecting the brain to the compound eyes. The antennal nerve developing from brain, extending towards antennal base. The suboesophageal complex is clearly narrower than the brain in frontal view, but in lateral view its maximum length is approximately twice the maximum length of the brain. But no ganglia in the ventral nerve cord are observed.

### Muscular system

A striated soft tissue structure is preserved on the sides and posterior edge of the interior of the head of the specimen. This structure starts at the root of the mandible and labrum, extending towards the posterior edge of the head and lower margin of pharyngeal gland (Fig. 2a, b). Based on its shape and position, it can be determined as the musculature of the head [32–35]. Some muscle tissues can be identified by comparison with the head muscles of living ants. M. crani-omandibularis internus (omd1) is the largest cephalic muscle, developing from the root of the mandibles, extending to the very back of the head, with clear signs of shrinkage; M. craniomandibularis externus (omd3) is lightly flattened triangular muscle developing at the inner base of the head, shrinkage; M. tentorioscapalis (ona) is the muscle attached to the root of the antanna; M. frontoepipharyngalis (olb2) is rather thick muscle, developing from the frontal area of the antennal base and synchronous attaching the base of labrum; M. pharyngoepipharyngealis (mpe) is very strongly developed longitudinal muscle, connecting the dorsal prepharyngeal wall and labral base. In addition, on both sides of the thoracic oesophagus, there are also distinctive remnants of flight muscles, distinctively shrinkage (Fig. 2d).

### Digestive system

In the head and mesosoma of YKLP-AMB-002, there is a duct-like tissue that connects to the mouthparts, passes through the brain and runs from the neck to the thorax of the specimen. Based on the shape and position, this tissue can be identified as the pharynx and oesophagus [32–34] (Fig. 2a, b). When viewed from the top, the pharynx is in a trapezoid shape–similar to its occurrence in living ants (maximum width: 231 μm, height: 104 μm). Beneath the pharynx, a plump spherical infrabuccal pocket is present (length: 134 μm, width: 174 μm, height: 91 μm). The pharynx is followed by an inflated oesophagus (maximum width: 271 μm, about 35% of the maximum head width, maximum height: 115 μm) passing through the middle of the brain and the head muscles, and eventually joins the thorax at the neck. In the mesosoma, the thoracic oesophagus is distinctively enlarged (maximum length: 657 μm, maximum height: 481 μm, i.e., nearly 75% of the height of thorax) (Fig. 2c, d). Due to the limitations of fossil preservation, there is no remnants of the abdominal oesophagus and the connection.

**Fig. 3:**
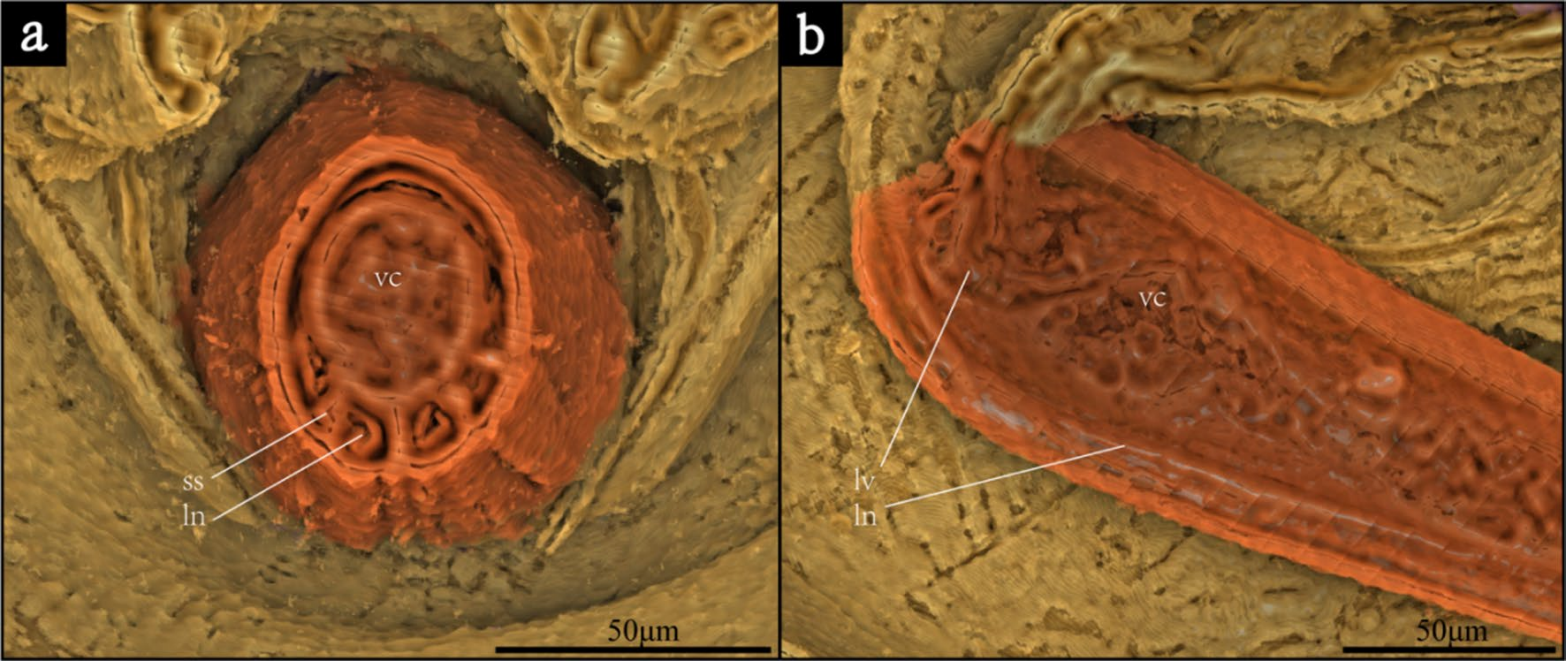
Sting anatomy. **a** Cross section of sting showing anatomy in dorsal view. **b** Longitudinal section of sting showing anatomy in lateral view. *ln* lancet; *lv* lancet valves; *ss* sting shaft; *vc* venom canal

In the gaster, a sharp and prominent sting is present (maximum width: 84 μm, total length: 1529 μm) (Fig. 2f), developing in the end of gaster. Inside the sting, the lancet, lancet valves and venom canal are well-preserved (Fig. 3). The external of the sting and the top posterior part of the abdomen cannot be shown in the CT data, but can be observed in optical photographs (Fig. 1a).

### Exocrine system

YKLP-AMB-002 also preserves the main exocrine glands, including mandibular glands, pharyngeal gland, labial glands, metapleural glands and venom gland. A pair of developed mandibular glands (width: 209 μm, length: 338 μm, the length means 69% of head length) connected with both mandible in the head. Though the left one is a bit residue, they are symmetrically arranged like living ants. Behind mandibular gland, a complete pharyngeal gland (width: 267 μm, length: 165 μm, thickness: 67 μm) connects with upper pharynx. In the head view, the pharyngeal gland shows a heart-shaped sac (Fig. 2a, b).

In the mesosoma, the membranous reservoir of labial gland (Fig. 2c, d) is preserved. It divides into left and right parts on the upper side of the thorax. The duct to the opening site was lost. Another one is the metapleural gland (Fig. 2c, e). A portion of soft tissue of the metapleural gland is recognized by the connection with its opening orifice (body surface). The gland itself was shown up in a symmetrical pair of irregularly spherical glands (width: 81 μm, height: 119 μm, thickness: 71 μm).

In the gaster, only an extremely developed venom gland (Fig. 2e, f) was preserved. The reservoir was distinctly inflated (Fig. 2f, g). The venom gland sac and the sting was connected by the main duct of poison sac (maximum height: 604 μm, i.e., 88% of the abdomen height, maximum width: 511 μm, i.e., 75% of abdomen width). Paired Cloacal chamber like structure (Hölldobler and Wilson, 1990) preserved in the end of abdomen.

**Fig. 4:**
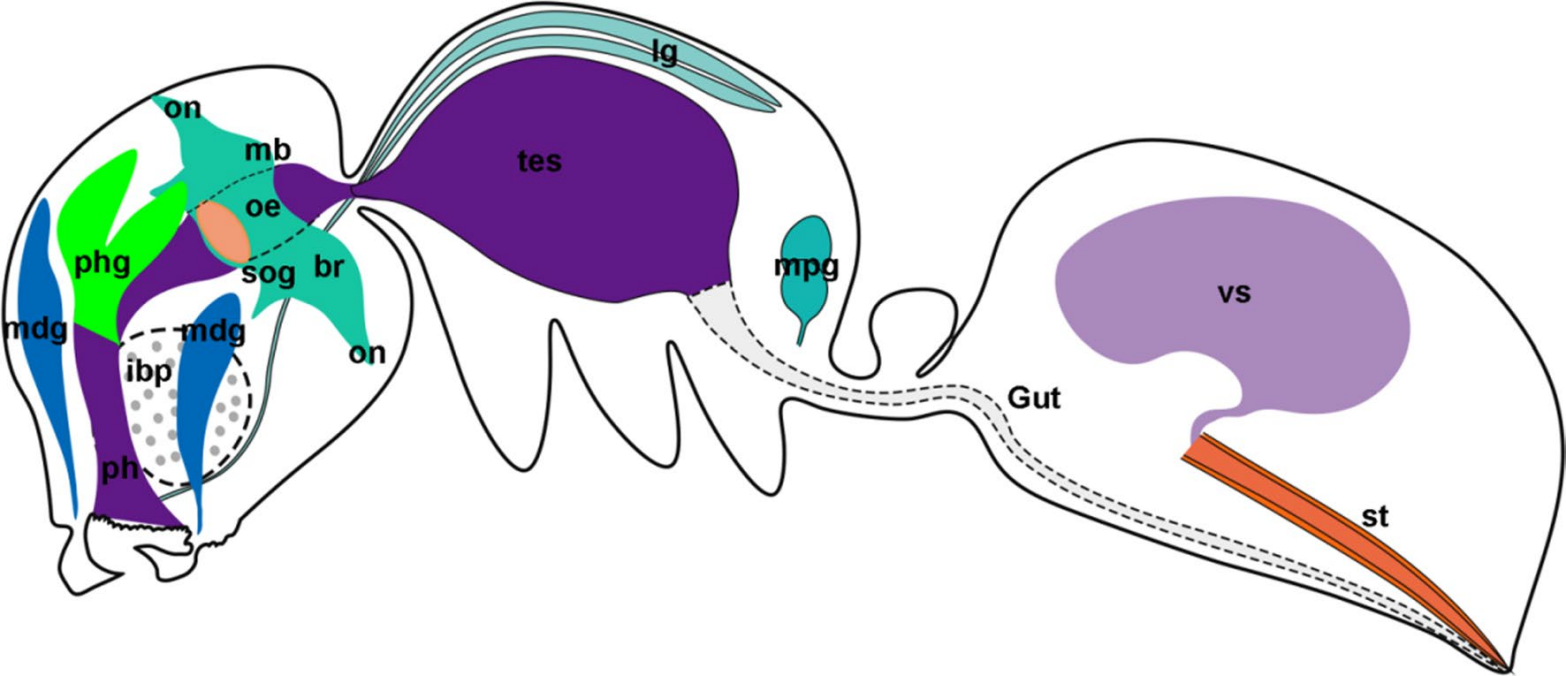
Diagram of internal organs of †*Zigrasimecia* sp. *br* brain; *ibp* infrabuccal pocket; *lg* labial gland; *mb* mushroom bodies; *mdg* mandibular gland; *mpg* metapleural gland; *oe* oesophagus; *on* optical nerve; *ph* pharynx; *phg* pharyngeal gland; *st* sting; *sog* suboesophageal ganglion; *tes* thoracic esophagus; *vs* venom gland sac

Based on the findings of the internal structure of the specimen, the internal structure pattern has been mapped for this ancient ant, ^†^*Zigrasimecia* (Fig. 4).

## Discussion

Fossils with soft-tissue preservation are rare, and often with only a certain part of the internal structures such as the muscular [17, 18, 21, 36–40], the neural [13, 14, 41–46], the cardiovascular [47], the glandular [13, 14, 21], the reproductive [19, 21, 48], or digestive [13, 14, 20] systems. Although the oldest fossil record of soft-tissue preservation can be traced back to the early Cambrian [41–43, 47], knowledge of the fossilization process of soft tissue in generally still remains rather limited [49], sometimes even controversial [50, 51]. Recently, the mineralization process of insects preserved in Kachin amber has been investigated and hypothesized—the resin prevented the degradation of the insects until it was cracked when buried in marine sediment [52]. Pore water with reactive chemical species of diagenesis then infiltrated the amber pieces through the cracks, which resulted in the calification or silification of the insect bodies [52]. On the one hand, this hypothesis explains why a great proportion of known amber inclusions are preserved in a solid state filled with minerals [16, 53]. On the other hand, it indicates that only a small number of amber pieces—most likely those least affected by chemical species of diagenesis—can preserve internal soft tissue. We summarized all the studies we know on the preservation of soft tissues within amber pieces, and noticed that almost every kind of internal organs of an arthropod can possibly carry have been reported (Additional file 1: Table S1). In a few cases, destructive methods such as sectioning combined with Transmission Electron Microscopy (TEM) even helped reveal cellular structures of the soft tissue [16, 38, 40, 53–55]. Notably, none of the previous studies on soft-tissue preservation in amber caught the complete internal organs of the animals. In the present study, we employed microscopic and computed tomographic techniques to reconstruct the exoskeleton and the almost complete internal organs of an ant—a gyne of ^†^*Zigrasimecia*—preserved in an amber piece (YKLP-AMB-002) from Kachin. We recovered the intact nervous, digestive, muscular and exocrine systems of the ant without destructing the sample (Figs. 1–4; Additional file 2: Video S1, Additional file 3: Video S2, Additional file 4: Video S3, Additional file 5: Video S4, Additional file 6: Video S5, Additional file 7: Video S6, Additional file 8: Video S7, Additional file 9: Video S8, Additional file 10: Video S9).

### Nervous system

Brain is an important organ for arthropods to control their activities including feeding, locomotion, and mating [56]. For social insects such as ants, their relatively large brain plays a key role in various actions such as communicating with other individuals from the same and/or different colonies [7, 57–59]. A brain and a sub esophageal ganglion are recovered from our specimen (Fig. 2a, b), suggesting that the gyne of ^†^*Zigrasimecia* bears a well-developed nervous system possibly for reproductive and brooding purposes [7].

Similar findings of a brain and a suboesophageal ganglion have been reported from a strepsipteran insect preserved in a Baltic amber piece [21]. Such anatomical characters are used to improve the understandings of the ground pattern situation of the entire Strepsiptera, and to further confirm the systematic position of †*Mengea* as the sister group of Strepsiptera [21]. Various population sizes, degrees of sociality and environments can lead to marked differences in the brain development and evolution of different ant groups [60]. In the present study, the proportion of the brain in the head of the gyne of ^†^*Zigrasimecia* is somewhere between that of the omnipotent ants [33, 35] and the more social groups such as *Formica rufa* [33]. The optic neuropils of this specimen look comparatively large, it may be related to the reproductive behavior of the gyne. However, further discussion of the size of ant brain at the species level is not possible at this stage, as the situation in other castes and worker specializations must be taken into account [57]. Future studies on the worker’s brain of ^†^*Zigrasimecia* should be explored when the material is available.

### Muscular system

Muscles seem to be the most commonly preserved soft tissue found in arthropod ambers (Additional file 1: Table S1). The best muscular preservation in ambers known so far probably comes from a dance fly in a Dominican amber pieces, with fine preservation of identifiable cellular structures such as myofibrils and densely packed mitochondria [38]. Muscle tissue has also been reported from Kachin ambers in the recent years. These include the dorsoventral flight muscles of a stinging wasp [17], the muscular bundles in the metathorax of a beetle [18], and those connecting the thorax and the abdomen of a stem ant [13, 14]. By contrast, muscles in the head of the animals are barely recovered.

The pattern of musculature in the head of extant ants is well studied [33–35, 61]. Those muscles play important roles in controlling the mouthparts during feeding and attacking processes. Our data allows us to carry out fine-scale identification of the head muscles in Cretaceous arthropods (Fig. 2). Similar to living ants, our specimen bears strong mandibular muscles that empowers predatory and carriage behaviors [34]. Both M. frontoepipharyngalis and M. pharyngoepipharyngealis of this fossil specimen are more likely robust than those of the Leptanillinae—a basal group of extant ants that catch preys [35]. Combined with the stout setae on the labrum (Fig. 1e, f), these powerful muscles may allow the animal to trap and feed on its prey with help from the armed labrum.

### Digestive system

Our specimen serves as an ideal model showing the feeding system of early ants—externally, it preserves the well-armed mouthparts (Fig. 1b, e, f) and an infrabuccal pocket sitting in the oral cavity whose width occupies one third of the head (Fig. 2b); internally, an inflated oesophagus runs through the head and extends the entire length of the body (The effect of deformation during petrification on the entire digestive tract cannot be excluded). Considering the relatively intact state of preservation (insignificant structural deformation in the vicinity of the esophagus), we can hypothesized reasonably that with such a unique feeding system, the Cretaceous ^†^*Zigrasimecia* could hunt on preys, absorb larger solid food debris than most living ants of similar body size [62, 63], and inject digestive enzymes from the labial gland, which usually opens at the base of the labium.

The crop, also called ‘social stomach’, is an important organ for the trophallaxis behaviour—food and/or information substance exchanging in the society of ants and many other eusocial animals [7]. The presence of a crop may be helpful in evaluating the society evolution level of the Cretaceous ants. However, the crop is not observable/or undeveloped in the gaster of our specimen (can not exclude fossilization artifact) (Fig. 2c, d), it may indicating that an undeveloped crop situation for the gyne of the Cretaceous ^†^*Zigrasimecia*, as seen in the queen of the modern ant *Monomorium pharaonis* [64]. The inflated oesophagus forms a crop-like structure in the thorax of our specimen, likely similar to the ‘thoracic crop’ [64–66] seen in two modern ants (*Pheidole aberrans* and *Pheidole deima*) [67] but bearing little evolutionary indication, in our view.

### Exocrine system

The exocrine system of ants plays important roles in many aspects such as alarm defense [11, 34] and pheromone secretion to attract mates during nuptial seasons [68]. Within the exocrine system, the metapleural gland is a diagnostic feature of ants and secrets volatiles as territory and nest-entrance marking, antisepsis, hygiene and chemical defense [69–71]. However, on fossil specimens, this gland is often only identified by its small opening on the exoskeleton of the posterior body [22, 72]. For instance, the openings of metapleural gland on the exoskeleton had been documented in both worker and reproductive castes of several fossil ants including ^†^*Zigrasimecia* [22–24, 26]*.* For the first time, we recover the inner structure of the metapleural gland in the fossil ants as a whole.

Our specimen also preserves a fossilized pharyngeal gland. The pharyngeal gland is present in almost all studied ant species [7] and is one of the main sources of colony odor by which ants can discriminate workers and queens, foragers and nurses, nestmates and enemies [73, 74]. Furthermore, the CT data shows a difference of this gland body with the most part of living ants, whose pharyngeal gland is usually glove-shaped [33, 75] with the fingers are extended inside the ants’ head, even cover most of the brain, but the fossil’s one is formed by a heart-shaped sac. In another word, the ant preserved in YKLP-AMB-002 already have the basic pharyngeal gland structure which may making effect to the social behavior of this ant group.

## Supplementary Information

**Additional file 1: Figure S1.** Artistic reconstruction of two alate females of ^†^*Zigrasimecia *sp. **Table S1.** Summary of studies related to the preservation of fossilized internal organs (present study marked in yellow).

**Additional file 2: Video S1.** 3D model of the thorax of ^†^*Zigrasimecia* sp. in lateral view.

**Additional file 3: Video S2.** 3D model of the thorax of ^†^*Zigrasimecia* sp. in dorsal view.

**Additional file 4: Video S3.** 3D model of the thorax of ^†^*Zigrasimecia* sp. in posterior view.

**Additional file 5: Video S4.** 3D model of the gaster of ^†^*Zigrasimecia* sp. in lateral view.

**Additional file 6: Video S5.** 3D model of the gaster of ^†^*Zigrasimecia* sp. in dorsal view.

**Additional file 7: Video S6.** 3D model of the gaster of ^†^*Zigrasimecia* sp. in posterior view.

**Additional file 8: Video S7.** 3D model of the head of ^†^*Zigrasimecia* sp. in lateral view.

**Additional file 9: Video S8.** 3D model of the head of ^†^*Zigrasimecia* sp. in frontal view.

**Additional file 10: Video S9.** 3D model of the head of ^†^*Zigrasimecia* sp. in top view.

## Data Availability

The datasets generated and/or analysed during the current study are available in the Zenodo repository (https://doi.org/10.5281/zenodo.7193141). Additional information supporting the results are provided as Additional file 2: Videos S1–Additional file 10: Videos S9. All specimens are kept at YKLP at Yunnan University. Photographic material of the studied material is available from the corresponding authors on request.
