# Supplementary material for: Unparalleled details of soft tissues in a Cretaceous ant
Source: BMC Ecol Evol. 2022 Dec 16;22:146. doi: 10.1186/s12862-022-02099-2 (PMC9756460; doi:10.1186/s12862-022-02099-2)
Supplement: Supplementary file 1 — Additional file 1: Figure S1. Artistic reconstruction of two alate females of †Zigrasimecia sp. Table S1. Summary of studies related to the preservation of fossilized internal organs (present study marked in yellow). [file 12862_2022_2099_MOESM1_ESM.pdf]

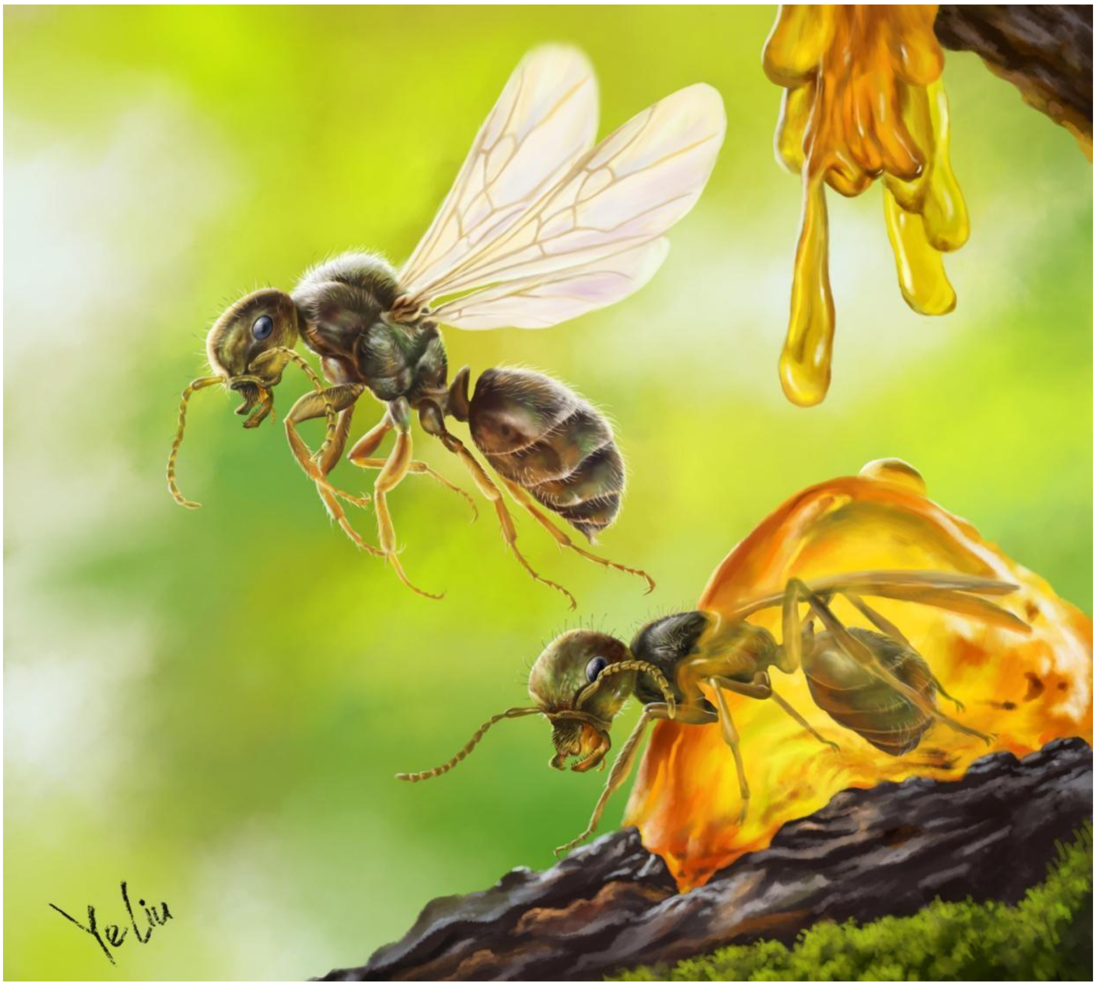

Figure S1. Artistic reconstruction of two alate females of †*Zigrasimecia* sp.

Table S1. Summary of studies related to the preservation of fossilized internal organs (present study marked in yellow).

Publications	Place of origin	Animal	Method	Internal structure				
				Nervous System	Digestive System	Muscular System	Exocrine System	Reproductive system
Present Study	Kachin	Ant	Micro-CT	√	√	√	√	√
Richter et al., 2022	Kachin	Ant	Micro-CT	√	√	√	√	
Boudinot et al., 2022	Kachin	Ant	Micro-CT	√	√	√	√	
Xing et al., 2021	Kachin	shrimp	Synchrotron and Micro-CT	√	√			
Li et al., 2021	Kachin	Coleoptera	Micro-CT			√		
Wang et al., 2020	Kachin	Ostracods	Micro-CT					√
Grimaldi et al., 2019	Kachin	wasp	Micro-CT			√		
Pohl et al., 2010	Baltic	Mengea	Synchrotron X-ray	√	√	√		√
Heethoff et al., 2009	Dominican	Oribatida	Synchrotron X-ray			√		√
Grimaldi et al., 1994	Dominican & Baltic	Hymenoptera, termites, beetles, gnats, flies	SEM & TEM	√		√		
Henwood, 1992a	Dominican	fly & beetles	SEM & TEM			√		
Henwood, 1992b	Dominican	coleopteran	SEM & TEM			√		
Poinar, 1992	Canadian	wasp	TEM			√		
Poinar and Hess, 1982	Baltic	gnats	TEM			√		
Poinar and Hess, 1985	Baltic	fly	TEM			√		
Mierzejewski, 1976	Baltic	fly	SEM	√				
Petrunkevitch, 1935	Baltic	fly	Microscope			√		
Kornilowitsch, 1903	Baltic	Diptera and Neuroptera	Microscope			√		

Video S1. 3D model of the thorax of †Zigrasimecia sp. in lateral view.
Video S2. 3D model of the thorax of †Zigrasimecia sp. in dorsal view.
Video S3. 3D model of the thorax of †Zigrasimecia sp. in posterior view.
Video S4. 3D model of the gaster of †Zigrasimecia sp. in lateral view.
Video S5. 3D model of the gaster of †Zigrasimecia sp. in dorsal view.
Video S6. 3D model of the gaster of †Zigrasimecia sp. in posterior view.
Video S7. 3D model of the head of †Zigrasimecia sp. in lateral view.
Video S8. 3D model of the head of †Zigrasimecia sp. in frontal view.
Video S9. 3D model of the head of †Zigrasimecia sp. in top view.
